# *catena*-Poly[(diaqua­strontium)-bis­{μ-5-[4-(1*H*-imidazol-1-yl)phen­yl]tetra­zolido}]

**DOI:** 10.1107/S1600536812013347

**Published:** 2012-03-31

**Authors:** Shao-Wei Tong, Shi-Jie Li, Wen-Dong Song, Dong-Liang Miao, Qi Deng

**Affiliations:** a College of Food Science and Technology, Guangdong Ocean University, Zhanjiang 524088, People’s Republic of China; b School of Environment Science and Engineering, Donghua University, Shanghai 200051, People’s Republic of China; c College of Science, Guangdong Ocean University, Zhanjiang 524088, People’s Republic of China

## Abstract

In the title complex polymer, [Sr(C_10_H_7_N_6_)_2_(H_2_O)_2_]_*n*_, the Sr^II^ atom lies on an inversion centre and is coordinated by four N atoms from two bidentate bridging *trans*-related 5-[4-(1*H*-imidazol-1-yl)phen­yl]tetra­zolide ligands [Sr—N = 2.387 (4) Å for the tetrazolide moiety and Sr—N = 2.273 (5) Å for the imidazole moiety], and by two O atoms from water mol­ecules [Sr—O = 2.464 (4) Å], giving a distorted octa­hedral coordination. Pairs of ligand bridges link the complex units, forming chains which extend along [111] and are inter-associated through O_water_—H⋯N hydrogen bonds, giving a two-dimensional network structure parallel to (001). Weak π–π stacking inter­actions between the benzene and imidazole rings are also present [minimum ring centroid separation = 3.691 (4) Å].

## Related literature

For our previous work on imidazole derivatives as ligands, see: Tong *et al.* (2011 ▶); Li *et al.* (2010 ▶). Wang *et al.* (2010 ▶). For related structures, see: Huang *et al.* (2009 ▶); Cheng (2011 ▶).
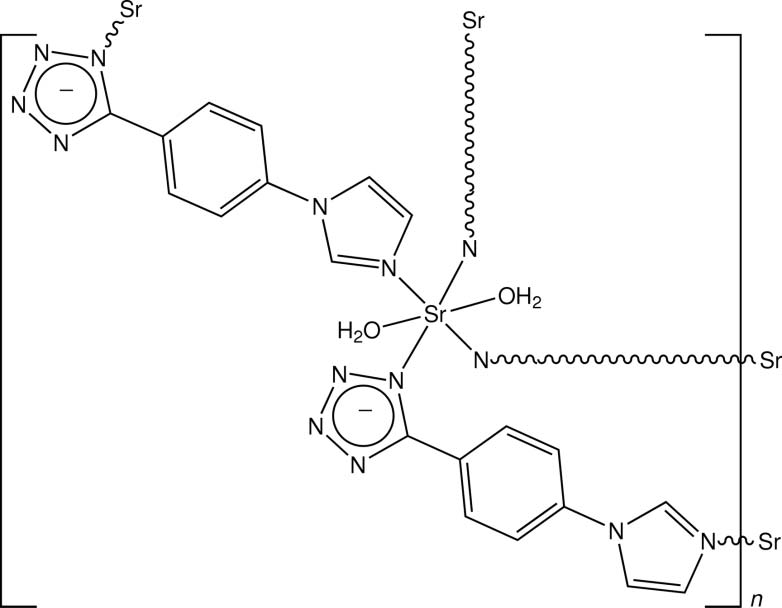


## Experimental

### 

#### Crystal data

[Sr(C_10_H_7_N_6_)_2_(H_2_O)_2_]
*M* *_r_* = 546.08
Triclinic, 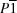

*a* = 7.6210 (6) Å
*b* = 8.0589 (7) Å
*c* = 9.1641 (9) Å
α = 102.783 (1)°
β = 97.544 (1)°
γ = 106.036 (2)°
*V* = 516.29 (8) Å^3^
*Z* = 1
Mo *K*α radiation
μ = 2.66 mm^−1^
*T* = 298 K
0.37 × 0.30 × 0.21 mm

#### Data collection

Bruker SMART CCD area-detector diffractometer
Absorption correction: multi-scan (*SADABS*; Bruker, 2007 ▶) *T*_min_ = 0.439, *T*_max_ = 0.605
2616 measured reflections
1789 independent reflections
1756 reflections with *I* > 2σ(*I*)
*R*_int_ = 0.013

#### Refinement

*R*[*F* ^2^ > 2σ(*F* ^2^)] = 0.036
*wR*(*F* ^2^) = 0.118
*S* = 1.17
1789 reflections
161 parameters
H-atom parameters constrained
Δρ_max_ = 0.79 e Å^−3^
Δρ_min_ = −0.43 e Å^−3^

### 

Data collection: *SMART* (Bruker, 2007 ▶); cell refinement: *SAINT* (Bruker, 2007 ▶); data reduction: *SAINT*; program(s) used to solve structure: *SHELXS97* (Sheldrick, 2008 ▶); program(s) used to refine structure: *SHELXL97* (Sheldrick, 2008 ▶); molecular graphics: *SHELXTL* (Sheldrick, 2008 ▶); software used to prepare material for publication: *SHELXTL*.

## Supplementary Material

Crystal structure: contains datablock(s) I, global. DOI: 10.1107/S1600536812013347/zs2191sup1.cif

Structure factors: contains datablock(s) I. DOI: 10.1107/S1600536812013347/zs2191Isup2.hkl

Additional supplementary materials:  crystallographic information; 3D view; checkCIF report

## Figures and Tables

**Table 1:** Hydrogen-bond geometry (Å, °)

*D*—H⋯*A*	*D*—H	H⋯*A*	*D*⋯*A*	*D*—H⋯*A*
O1—H1*C*⋯N1^i^	0.85	2.07	2.915 (7)	171
O1—H1*D*⋯N2^ii^	0.85	2.10	2.948 (6)	171

Symmetry codes: (i) 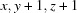; (ii) 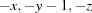.

**Fig. 1.**
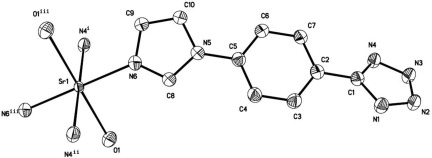
Molecular configuration and atom numbering scheme for the title complex showing 30% probability ellipsoids. For symmetry codes: (i) x + 1, y + 1, z + 1; (ii) -x, -y, -z; (iii) -x + 1, -y + 1, -z + 1.

## supplementary crystallographic information

### Comment

Recently, our research group has shown great interest in the solid-state coordination chemistry of *N*-heterocyclic carboxylic acids, such as 2-propyl-1*H*-imidazole-4,5-dicarboxylic acid and 1*H*-benzimidazole-5,6-dicarboxylic acid. We have synthesized a number of metal complexes using the monoanionic 5-[(4-imidazol-1-yl)phenyl]tetrazolide ligand with a series of metals, e.g. Mn, Cd and Sr (Tong *et al.*, 2011; Li *et al.*, 2010; Wang *et al.*, 2010). In this paper, we report the structure of a new strontium complex with this ligand, obtained under hydrothermal conditions, the title complex polymer, [Sr(C_10_H_7_N_6_)_2_(H_2_O)_2_]_n_. The centrosymmetric complex molecule (Fig. 1) comprises a Sr^II^ ion coordinated by four N atoms from the two bidentate bridging *trans*- related 5-[(4-imidazol-1-yl)phenyl]tetrazolido ligands (two tetrazole and two imidazole) and two O atoms from the water molecules, giving a distorted octahedral stereochemistry [Sr—N = 2.273 (4), 2.387 (5) Å and Sr—O = 2.464 (4) Å]. Duplex bridging ligand molecules link the complex molecules forming polymer chains which extend along [111] and are inter-associated through water O—H···N hydrogen bonds (Table 1) giving a two-dimensional network structure. Weak π–π stacking interactions are also present between the phenyl and the imidazole rings [minimum ring centroid separation, 3.691 (4) Å]. The structures of similar complexes are also known (Huang *et al.*, 2009; Cheng, 2011).

### Experimental

A mixture of strontium chloride (0.1 mmol, 0.027 g) and 5-[4-imidazol-1-yl)phenyl]tetrazole (0.2 mmol, 0.043 g) in 12 ml of water was sealed in an autoclave equipped with a Teflon liner (25 ml) and then heated at 413 K for 3 days. Crystals of the title compound were obtained by slow evaporation of the solvent at room temperature.

### Refinement

H atoms of the water molecule were located in a difference-Fourier map and refined as riding with an O—H distance restraint of 0.85 Å, with *U*_iso_(H) = 1.2 *U*_eq_(O). The imidazolyl and phenyl H atoms were located in a difference-Fourier but were refined as riding with C—H = 0.93 Å also with *U*_iso_(H) = 1.2*U*_eq_(C).

### Crystal data

**Table:**

[Sr(C_10_H_7_N_6_)_2_(H_2_O)_2_]	*Z* = 1
*M**_r_* = 546.08	*F*(000) = 276
Triclinic, *P*1	*D*_x_ = 1.756 Mg m^−^^3^
Hall symbol: -P 1	Mo *K*α radiation, λ = 0.71073 Å
*a* = 7.6210 (6) Å	Cell parameters from 2869 reflections
*b* = 8.0589 (7) Å	θ = 2.8–28.3°
*c* = 9.1641 (9) Å	µ = 2.66 mm^−^^1^
α = 102.783 (1)°	*T* = 298 K
β = 97.544 (1)°	Block, colourless
γ = 106.036 (2)°	0.37 × 0.30 × 0.21 mm
*V* = 516.29 (8) Å^3^	

### Data collection

**Table:**

Bruker SMART CCD area-detector diffractometer	1789 independent reflections
Radiation source: fine-focus sealed tube	1756 reflections with *I* > 2σ(*I*)
Graphite monochromator	*R*_int_ = 0.013
φ and ω scans	θ_max_ = 25.0°, θ_min_ = 2.7°
Absorption correction: multi-scan (*SADABS*; Bruker, 2007)	*h* = −9→9
*T*_min_ = 0.439, *T*_max_ = 0.605	*k* = −9→7
2616 measured reflections	*l* = −10→9

### Refinement

**Table:**

Refinement on *F*^2^	Secondary atom site location: difference Fourier map
Least-squares matrix: full	Hydrogen site location: inferred from neighbouring sites
*R*[*F*^2^ > 2σ(*F*^2^)] = 0.036	H-atom parameters constrained
*wR*(*F*^2^) = 0.118	*w* = 1/[σ^2^(*F*_o_^2^) + (0.0473*P*)^2^ + 1.6581*P*] where *P* = (*F*_o_^2^ + 2*F*_c_^2^)/3
*S* = 1.17	(Δ/σ)_max_ < 0.001
1789 reflections	Δρ_max_ = 0.79 e Å^−^^3^
161 parameters	Δρ_min_ = −0.43 e Å^−^^3^
0 restraints	Extinction correction: *SHELXL97* (Sheldrick, 2008), Fc^*^=kFc[1+0.001xFc^2^λ^3^/sin(2θ)]^-1/4^
Primary atom site location: structure-invariant direct methods	Extinction coefficient: 0.260 (15)

### Special details

**Table:**

Geometry. Bond distances, angles etc. have been calculated using the rounded fractional coordinates. All su's are estimated from the variances of the (full) variance-covariance matrix. The cell esds are taken into account in the estimation of distances, angles and torsion angles
Refinement. Refinement of *F*^2^ against ALL reflections. The weighted *R*-factor *wR* and goodness of fit *S* are based on *F*^2^, conventional *R*-factors *R* are based on *F*, with *F* set to zero for negative *F*^2^. The threshold expression of *F*^2^ > σ(*F*^2^) is used only for calculating *R*-factors(gt) *etc*. and is not relevant to the choice of reflections for refinement. *R*-factors based on *F*^2^ are statistically about twice as large as those based on *F*, and *R*- factors based on ALL data will be even larger.

### Fractional atomic coordinates and isotropic or equivalent isotropic displacement parameters (Å^2^)

**Table:**

	*x*	*y*	*z*	*U*_iso_*/*U*_eq_	
Sr1	0.50000	0.50000	0.50000	0.0271 (2)	
O1	0.3110 (6)	0.2631 (5)	0.5968 (5)	0.0511 (12)	
N1	−0.0566 (7)	−0.7449 (6)	−0.3419 (5)	0.0468 (16)	
N2	−0.2033 (7)	−0.8777 (6)	−0.4402 (6)	0.0478 (16)	
N3	−0.3273 (6)	−0.8089 (6)	−0.4930 (6)	0.0480 (16)	
N4	−0.2656 (6)	−0.6290 (6)	−0.4306 (5)	0.0451 (14)	
N5	0.3031 (6)	0.1033 (6)	0.0479 (5)	0.0411 (12)	
N6	0.4352 (6)	0.3262 (6)	0.2555 (5)	0.0435 (14)	
C1	−0.0992 (7)	−0.5942 (7)	−0.3381 (6)	0.0402 (17)	
C2	0.0161 (7)	−0.4150 (7)	−0.2417 (6)	0.0418 (17)	
C3	0.1352 (8)	−0.3925 (8)	−0.1057 (7)	0.0477 (17)	
C4	0.2324 (8)	−0.2232 (7)	−0.0114 (7)	0.0481 (17)	
C5	0.2105 (7)	−0.0735 (7)	−0.0516 (6)	0.0403 (17)	
C6	0.0955 (8)	−0.0935 (7)	−0.1875 (6)	0.0473 (17)	
C7	−0.0003 (8)	−0.2634 (7)	−0.2825 (6)	0.0454 (17)	
C8	0.3753 (8)	0.1501 (7)	0.1989 (6)	0.0433 (17)	
C9	0.4015 (8)	0.3956 (7)	0.1358 (6)	0.0460 (17)	
C10	0.3208 (8)	0.2604 (7)	0.0071 (6)	0.0475 (17)	
H1C	0.20900	0.27310	0.61930	0.0610*	
H1D	0.29230	0.15410	0.55030	0.0610*	
H3	0.14980	−0.49270	−0.07760	0.0580*	
H4	0.31260	−0.21000	0.07910	0.0570*	
H6	0.08210	0.00710	−0.21560	0.0570*	
H7	−0.07670	−0.27600	−0.37480	0.0540*	
H8	0.38200	0.06900	0.25590	0.0520*	
H9	0.42970	0.51710	0.14180	0.0550*	
H10	0.28420	0.27160	−0.09020	0.0570*	

### Atomic displacement parameters (Å^2^)

**Table:**

	*U*^11^	*U*^22^	*U*^33^	*U*^12^	*U*^13^	*U*^23^
Sr1	0.0303 (4)	0.0239 (4)	0.0238 (4)	0.0093 (2)	0.0006 (2)	0.0018 (2)
O1	0.049 (2)	0.043 (2)	0.061 (2)	0.0155 (17)	0.0138 (19)	0.0107 (18)
N1	0.046 (3)	0.043 (2)	0.050 (3)	0.016 (2)	0.007 (2)	0.009 (2)
N2	0.049 (3)	0.036 (2)	0.054 (3)	0.013 (2)	0.007 (2)	0.006 (2)
N3	0.044 (3)	0.038 (2)	0.057 (3)	0.012 (2)	0.004 (2)	0.008 (2)
N4	0.044 (2)	0.036 (2)	0.049 (3)	0.0105 (19)	0.004 (2)	0.0048 (19)
N5	0.045 (2)	0.040 (2)	0.037 (2)	0.0126 (19)	0.0065 (19)	0.0099 (18)
N6	0.048 (3)	0.039 (2)	0.039 (2)	0.012 (2)	0.0053 (19)	0.0061 (19)
C1	0.039 (3)	0.041 (3)	0.041 (3)	0.015 (2)	0.008 (2)	0.009 (2)
C2	0.039 (3)	0.044 (3)	0.042 (3)	0.014 (2)	0.010 (2)	0.009 (2)
C3	0.049 (3)	0.042 (3)	0.051 (3)	0.018 (2)	0.001 (3)	0.011 (2)
C4	0.049 (3)	0.046 (3)	0.045 (3)	0.017 (2)	−0.002 (2)	0.008 (2)
C5	0.040 (3)	0.042 (3)	0.038 (3)	0.012 (2)	0.010 (2)	0.009 (2)
C6	0.055 (3)	0.039 (3)	0.044 (3)	0.010 (2)	0.004 (2)	0.013 (2)
C7	0.048 (3)	0.044 (3)	0.038 (3)	0.009 (2)	0.003 (2)	0.009 (2)
C8	0.051 (3)	0.039 (3)	0.039 (3)	0.015 (2)	0.005 (2)	0.010 (2)
C9	0.056 (3)	0.039 (3)	0.042 (3)	0.013 (2)	0.008 (2)	0.013 (2)
C10	0.061 (3)	0.041 (3)	0.038 (3)	0.013 (3)	0.006 (2)	0.012 (2)

### Geometric parameters (Å, º)

**Table:**

Sr1—O1	2.464 (4)	N6—C9	1.363 (7)
Sr1—N6	2.273 (4)	C1—C2	1.473 (8)
Sr1—N4^i^	2.387 (5)	C2—C7	1.387 (8)
Sr1—N4^ii^	2.387 (5)	C2—C3	1.386 (8)
Sr1—O1^iii^	2.464 (4)	C3—C4	1.382 (9)
Sr1—N6^iii^	2.273 (4)	C4—C5	1.382 (8)
O1—H1C	0.8500	C5—C6	1.375 (8)
O1—H1D	0.8500	C6—C7	1.385 (8)
N1—N2	1.362 (7)	C9—C10	1.352 (8)
N1—C1	1.336 (8)	C3—H3	0.9300
N2—N3	1.313 (7)	C4—H4	0.9300
N3—N4	1.355 (7)	C6—H6	0.9300
N4—C1	1.350 (7)	C7—H7	0.9300
N5—C8	1.347 (7)	C8—H8	0.9300
N5—C10	1.375 (7)	C9—H9	0.9300
N5—C5	1.435 (7)	C10—H10	0.9300
N6—C8	1.321 (7)		
			
O1—Sr1—N6	94.69 (16)	N1—C1—N4	110.9 (5)
O1—Sr1—N4^i^	81.30 (16)	N4—C1—C2	124.4 (5)
O1—Sr1—N4^ii^	98.70 (16)	N1—C1—C2	124.7 (5)
O1—Sr1—O1^iii^	180.00	C3—C2—C7	118.4 (5)
O1—Sr1—N6^iii^	85.31 (16)	C1—C2—C7	119.8 (5)
N4^i^—Sr1—N6	90.56 (16)	C1—C2—C3	121.7 (5)
N4^ii^—Sr1—N6	89.44 (16)	C2—C3—C4	120.9 (6)
O1^iii^—Sr1—N6	85.31 (16)	C3—C4—C5	120.0 (6)
N6—Sr1—N6^iii^	180.00	N5—C5—C4	121.0 (5)
N4^i^—Sr1—N4^ii^	180.00	N5—C5—C6	119.2 (5)
O1^iii^—Sr1—N4^i^	98.70 (16)	C4—C5—C6	119.8 (5)
N4^i^—Sr1—N6^iii^	89.44 (16)	C5—C6—C7	120.0 (5)
O1^iii^—Sr1—N4^ii^	81.30 (16)	C2—C7—C6	120.9 (5)
N4^ii^—Sr1—N6^iii^	90.56 (16)	N5—C8—N6	111.2 (5)
O1^iii^—Sr1—N6^iii^	94.69 (16)	N6—C9—C10	109.4 (5)
H1C—O1—H1D	108.00	N5—C10—C9	106.7 (5)
Sr1—O1—H1D	119.00	C2—C3—H3	120.00
Sr1—O1—H1C	118.00	C4—C3—H3	120.00
N2—N1—C1	105.0 (5)	C3—C4—H4	120.00
N1—N2—N3	109.8 (5)	C5—C4—H4	120.00
N2—N3—N4	108.9 (5)	C5—C6—H6	120.00
N3—N4—C1	105.5 (5)	C7—C6—H6	120.00
Sr1^iv^—N4—N3	110.5 (3)	C2—C7—H7	120.00
Sr1^iv^—N4—C1	143.5 (4)	C6—C7—H7	120.00
C8—N5—C10	106.5 (5)	N5—C8—H8	124.00
C5—N5—C8	128.0 (5)	N6—C8—H8	124.00
C5—N5—C10	125.3 (4)	N6—C9—H9	125.00
Sr1—N6—C8	131.1 (4)	C10—C9—H9	125.00
Sr1—N6—C9	120.4 (4)	N5—C10—H10	127.00
C8—N6—C9	106.2 (4)	C9—C10—H10	127.00
			
O1—Sr1—N6—C8	−20.5 (5)	C10—N5—C8—N6	0.7 (7)
O1—Sr1—N6—C9	139.5 (4)	C5—N5—C10—C9	174.7 (5)
N4^i^—Sr1—N6—C8	60.8 (5)	C8—N5—C10—C9	−0.6 (7)
N4^i^—Sr1—N6—C9	−139.2 (4)	Sr1—N6—C8—N5	161.7 (4)
N4^ii^—Sr1—N6—C8	−119.2 (5)	C9—N6—C8—N5	−0.5 (7)
N4^ii^—Sr1—N6—C9	40.8 (4)	Sr1—N6—C9—C10	−164.3 (4)
O1^iii^—Sr1—N6—C8	159.5 (5)	C8—N6—C9—C10	0.1 (7)
O1^iii^—Sr1—N6—C9	−40.5 (4)	N1—C1—C2—C3	−26.5 (9)
C1—N1—N2—N3	0.3 (6)	N1—C1—C2—C7	156.8 (6)
N2—N1—C1—N4	−0.3 (6)	N4—C1—C2—C3	151.0 (6)
N2—N1—C1—C2	177.6 (5)	N4—C1—C2—C7	−25.6 (8)
N1—N2—N3—N4	−0.1 (6)	C1—C2—C3—C4	−175.4 (6)
N2—N3—N4—C1	−0.1 (6)	C7—C2—C3—C4	1.3 (9)
N2—N3—N4—Sr1^iv^	−173.6 (4)	C1—C2—C7—C6	174.8 (5)
N3—N4—C1—N1	0.2 (6)	C3—C2—C7—C6	−2.0 (9)
N3—N4—C1—C2	−177.6 (5)	C2—C3—C4—C5	0.5 (9)
Sr1^iv^—N4—C1—N1	170.0 (4)	C3—C4—C5—N5	177.0 (5)
Sr1^iv^—N4—C1—C2	−7.8 (10)	C3—C4—C5—C6	−1.7 (9)
C8—N5—C5—C4	−19.4 (9)	N5—C5—C6—C7	−177.7 (5)
C8—N5—C5—C6	159.3 (6)	C4—C5—C6—C7	1.0 (9)
C10—N5—C5—C4	166.4 (6)	C5—C6—C7—C2	0.8 (9)
C10—N5—C5—C6	−14.9 (8)	N6—C9—C10—N5	0.3 (7)
C5—N5—C8—N6	−174.4 (5)		

Symmetry codes: (i) *x*+1, *y*+1, *z*+1; (ii) −*x*, −*y*, −*z*; (iii) −*x*+1, −*y*+1, −*z*+1; (iv) *x*−1, *y*−1, *z*−1.

### Hydrogen-bond geometry (Å, º)

**Table:**

*D*—H···*A*	*D*—H	H···*A*	*D*···*A*	*D*—H···*A*
O1—H1*C*···N1^v^	0.85	2.07	2.915 (7)	171
O1—H1*D*···N2^vi^	0.85	2.10	2.948 (6)	171

Symmetry codes: (v) *x*, *y*+1, *z*+1; (vi) −*x*, −*y*−1, −*z*.
